# Influences of Gestational Obesity on Associations between Genotypes and Gene Expression Levels in Offspring following Maternal Gastrointestinal Bypass Surgery for Obesity

**DOI:** 10.1371/journal.pone.0117011

**Published:** 2015-01-20

**Authors:** Frédéric Guénard, Maxime Lamontagne, Yohan Bossé, Yves Deshaies, Katherine Cianflone, John G. Kral, Picard Marceau, Marie-Claude Vohl

**Affiliations:** 1 Institute of Nutrition and Functional Foods (INAF) and Department of Food Science and Nutrition, Laval University, Quebec, Canada; 2 Endocrinology and Nephrology, CHU de Quebec Research Center, Quebec, Canada; 3 Quebec Heart and Lung Institute, Quebec, Canada; 4 Department of Molecular Medicine, Laval University, Quebec, Canada; 5 Department of Medicine, Laval University, Quebec, Canada; 6 Department of Surgery, SUNY Downstate Medical Center, Brooklyn, New York, United States of America; 7 Department of Surgery, Laval University, Quebec, Canada; IRCCS Scientific Institute and Regional General Hospital Casa Sollievo della Sofferenza Opera di Padre Pio da Pietrelcina, ITALY

## Abstract

Maternal obesity and excess gestational weight gain with compromised metabolic fitness predispose offspring to lifelong obesity and its comorbidities. We demonstrated that compared to offspring born before maternal gastrointestinal bypass surgery (BMS) those born after (AMS) were less obese, with less cardiometabolic risk reflected in the expression and methylation of diabetes, immune and inflammatory pathway genes. Here we examine relationships between gestational obesity and offspring gene variations on expression levels.

**Methods:**

Whole-genome genotyping and gene expression analyses in blood of 22 BMS and 23 AMS offspring from 19 mothers were conducted using Illumina HumanOmni-5-Quad and HumanHT-12 v4 Expression BeadChips, respectively. Using PLINK we analyzed interactions between offspring gene variations and maternal surgical status on offspring gene expression levels. Altered biological functions and pathways were identified and visualized using DAVID and Ingenuity Pathway Analysis.

**Results:**

Significant interactions (p ≤ 1.22x10^-12^) were found for 525 among the 16,060 expressed transcripts: 1.9% of tested SNPs were involved. Gene function and pathway analysis demonstrated enrichment of transcription and of cellular metabolism functions and overrepresentation of cellular stress and signaling, immune response, inflammation, growth, proliferation and development pathways.

**Conclusion:**

We suggest that impaired maternal gestational metabolic fitness interacts with offspring gene variations modulating gene expression levels, providing potential mechanisms explaining improved cardiometabolic risk profiles of AMS offspring related to ameliorated maternal lipid and carbohydrate metabolism.

## Introduction

Epidemiological studies demonstrate that parental obesity increases obesity risk in offspring and suggest an important role of the intrauterine environment owing to stronger associations between maternal than paternal body mass index (BMI) with offspring obesity [1, 2]. Maternal obesity, excess gestational weight gain, high inter-pregnancy BMI and gestational diabetes increase risks of offspring obesity, type 2 diabetes mellitus (T2DM), cardiovascular disease (CVD) and fatty liver [3–5]. Environmental and genetic factors mediate the link between parental obesity and increased risk of obesity in offspring [2, 6]; family and twin studies demonstrate heritability of obesity and CVD risk factors [7, 8].

Large-scale genome-wide association studies (GWAS) have consistently revealed the presence of specific genes in metabolic diseases such as type 1 and T2DM [9, 10] and obesity [7, 11]. GWAS on expression traits identified variations regulating gene expression (expression quantitative trait loci; eQTL) and demonstrated that gene expression levels show complex inheritance patterns [12, 13]. Such studies elucidate basic processes of gene regulation and may identify the pathogenesis of prevalent diseases adding information to associations identified by GWAS.

Gene expression levels are greatly affected by genetic and environmental factors [14] where gene variations have the potential to attenuate or amplify environmental effects. An adverse intrauterine environment has long been known to contribute to metabolic and cardiovascular diseases [15] where differences in expression levels between offspring born under different maternal conditions were reported for specific genes and at genome-wide level [16–19]. Several loci associated with specific traits interact with intrauterine environment [20–22]. A striking example of such gene-environment interaction is the association of *SIRT1* SNPs with lower prevalence of type 2 diabetes observed in individuals prenatally exposed to famine *in utero* but not in those not exposed to famine.

Bariatric bypass operations improve glucose and lipid metabolism and treat and/or prevent hypertension, dyslipidemia, T2DM and fatty liver disease [23–25]. Similar to weight loss [26, 27], bariatric surgery results in changes in gene expression levels [28, 29]. Our studies uniquely demonstrated that offspring born after maternal gastrointestinal bypass surgery (AMS) exhibit lower prevalence of severe obesity, greater insulin sensitivity and improved lipid profiles compared to offspring born before maternal surgery (BMS) [30, 31]. Recently, we demonstrated that these improvements are associated with differences in gene expression and methylation of genes involved in diabetes and immune and inflammatory pathways [17, 32].

In order to further explore the role of the intrauterine environment in the determination of offspring phenotype and to provide molecular mechanisms explaining changes in cardiometabolic risk markers of AMS vs. BMS offspring, we studied the combined influence of maternal surgical status and offspring gene variations on offspring gene expression levels.

## Materials and Methods

### Subjects

Women from Quebec City and surrounding areas (administrative regions of Capitale-Nationale, Mauricie and Chaudière-Appalaches) who had given birth before and after biliopancreatic diversion with duodenal switch [25] for severe obesity were eligible. We recruited a subset of 19 unrelated mothers aged 34–51 years having offspring aged 2–23 years, 22 born before and 23 after maternal operations. Between July and October 2010 mothers and offspring visited the Quebec Heart and Lung Institute (Quebec City, Quebec, Canada) or a regional hospital for clinical evaluation and blood sampling. There were 15 mothers with siblings born before and after surgery (21 BMS and 18 AMS), one with BMS offspring only (1 BMS) and 3 mothers with only AMS offspring (5 AMS).

Maternal pre-surgical data were obtained from medical records. At the office visit weight and percent body fat were determined for individuals aged 6 years or more (BMS, N = 21; AMS, N = 15) using bioelectric impedance analysis (Tanita; Arlington Heights, IL). Height and resting systolic (SBP) and diastolic (DBP) blood pressure were obtained using standardized procedures. BMI was calculated for mothers and adults and BMI percentiles for children were obtained from the National Health and Nutrition Examination Survey 2000 chart [33]. BMI Z-score was calculated for children using charts from the Centers for Disease Control and Prevention [34]. Fasting whole blood samples were collected from an antecubital vein into tubes containing EDTA and PAXgene Blood RNA collection tubes (Qiagen, Valencia, CA, USA). Plasma lipid, glucose and insulin concentrations were measured as previously described [35]. Lipid and glucose levels values from 3 AMS non-fasting offspring were excluded. The homeostatic model assessment of insulin resistance (HOMA-IR) index was calculated as fasting glucose x insulin/22.5. Levels of high-sensitivity C-reactive protein (CRP) were measured with a BN ProSpec nephelometer (Siemens Canada Limited, Oakville, Ontario, Canada) [36]. CRP values under the detection limit (< 0.17 mg/L) were arbitrarily set at detection limit.

### Ethics Statement

This study was approved by the Quebec Heart and Lung Institute Ethics Committee. Written informed consent was obtained from mothers and adult offspring and assent from minor offspring were obtained from mothers.

### Gene expression analysis

Gene expression levels of the 45 offspring analyzed here were obtained from previous studies from our group evaluating differences in gene expression and methylation of genes involved in diabetes, immune and inflammatory pathways [17, 32]. Briefly, total RNA was isolated and purified from offspring whole blood using PAXgene Blood RNA Kit (Qiagen). The quality and integrity of the purified RNA was assessed using both the NanoDrop (Thermo Scientific, Wilmington, DE, USA) and the 2100 Bioanalyzer (Agilent Technologies, Cedar Creek, TX, USA). Expression levels were measured using the HumanHT-12 v4 Expression BeadChip (Illumina Inc., San Diego, CA) with 250 ng of total RNA and processed at the McGill University and Genome Quebec Innovation Centre (Montreal, Canada). Expression data were visualized and analyzed using the FlexArray software [37] (version 1.6) and the lumi R package was used for expression data analysis and normalization. To be considered as expressed, a probe had to show a detection p-value ≤ 0.05 in at least 25% of samples of a group. Among the 47,323 probes on the microarray, 16,060 (33.9%) showed significant gene expression in blood and were used as dependent expression phenotypes (expression traits) for analysis of interactions between offspring gene variations and maternal obesity status (GEO accession number GSE44407).

### DNA extraction and genome-wide genotyping

Genomic DNA was isolated from offspring blood buffy coat using the GenElute Blood Genomic DNA kit (Sigma, St Louis, MO, USA). Quantification and verification of DNA quality were conducted via both NanoDrop spectrophotometer (Thermo Scientific, Wilmington, DE, USA) and PicoGreen DNA methods. Genotyping was performed at McGill University and Genome Quebec Innovation Centre (Montreal, Canada) using Illumina HumanOmni-5-Quad BeadChip (Illumina Inc., San Diego, CA), according to the manufacturer’s instructions. Each HumanOmni-5-Quad BeadChip contained 4,301,331 markers.

### SNPs and sample quality control

Calculations of allele frequencies and tests of SNP data for Hardy-Weinberg equilibrium (HWE) were performed using PLINK [38] (version 1.07). Standard quality control exclusion criteria for the SNPs were used: call rate < 95%, genotype distribution deviating from Hardy-Weinberg Equilibrium (p-values less than 10^–7^) and monomorphic SNPs or those with a minor allele frequency (MAF) < 0.01 [39]. A total of 1,751,034 SNPs were excluded leaving 2,550,297 SNPs for statistical analyses. All samples were tested for call rate (> 90%), ethnicity (Caucasian; HapMap) and gender mismatch based on genotyping data. No subjects were excluded: all 45 samples were used in further analysis.

### Statistical analysis

Anthropometric and clinical data were expressed as mean ± SD. Maternal treatment effect on anthropometric-, blood pressure-, lipid profile- and glucose-related variables was assessed using a within-subject, paired t-test. Differences between BMS and AMS offspring were tested using analysis of variance (general linear model, type III sum of squares) and adjusted for the effects of sex and puberty. BMI percentile and BMI Z-score being obtained from age- and sex-specific charts, no further adjustments for age and sex were made to test for differences between BMS and AMS offspring for those adiposity measurements. Severe obesity in offspring was defined as BMI percentile > 98% and Z-score > 3. Transformations were applied to non-normally distributed variables (log10 transformed for insulin and HOMA-IR; negative inverse transformed for C-reactive protein). In the absence of Tanner scores, we arbitrarily defined puberty as 12 years for female and 14 years for male offspring based on Canadian sex-specific probabilities of having entered puberty [40]. Differences in severe obesity between BMS and AMS offspring were evaluated using BMI percentile and BMI Z-score and tested using Fisher’s exact test. P-values for CRP were adjusted for the effects of sex, puberty and BMI percentile. Statistical analyses were done using the SAS software version 9.2 (SAS Institute Inc). Statistical significance was defined as p ≤ 0.05. Interactions between offspring gene variations and maternal surgical status were tested on offspring gene expression levels in whole blood using PLINK. Differences in regression slopes obtained from additive model were then tested between BMS and AMS offspring. Bonferroni correction was applied to correct for multiple testing of offspring gene variation x environment interactions thus leading to a p-value cutoff of p ≤ 1.22x10–^12^ (as calculated with 0.05/ (2,550,297 SNPs x 16,060 transcripts)) to claim statistical significance. Linkage disequilibrium (LD; r^2^) between SNPs demonstrating significant interactions was calculated using Haploview [41] to assess the number of independent (non-linked) SNPs. The tagger algorithm implemented in Haploview was used to identify tag SNPs among the significant polymorphisms (r^2^ threshold = 0.8).

### Gene functions and pathways analysis

Two independent function and pathway analysis tools were employed to identify potentially enriched functions and overrepresented pathways from the list of transcripts demonstrating significant interactions, namely the Database for Annotation, Visualization, and Integrated Discovery (DAVID; http://david.abcc.ncifcrf.gov) bioinformatics resources [42, 43] and Ingenuity Pathway Analysis (IPA). DAVID provided annotation for the list of transcripts and computed annotation term enrichment to highlight the most relevant functions from the list of transcripts. Similarly, IPA classified each transcript from this list according to function and pathway. Using a right-tailed Fisher’s exact test, IPA measured the likelihood that transcripts from the list participate in each function/pathway solely due to chance and calculated p-values. Enriched functions and overrepresented pathways were then obtained from transcripts showing interactions.

## Results

### Characteristics of 19 mothers and their 45 offspring

Mean postoperative follow-up for the mothers after bilio-pancreatic diversion surgery was 12 years 7 months (range: 4 years 11 months to 22 years 4 months). Preoperative weight was 121.6 ± 18.7 kg (BMI = 45.1 ± 7.4) and 74.9 ± 12.2 kg (BMI = 27.6 ± 4.9) at follow-up, a mean loss of 46.7 kg, associated with significant, clinically important improvements in fasting plasma lipids (p ≤ 0.005 for TG, HDL-C, LDL-C, total-C and total-C/HDL-C ratio), glucose levels (5.81 ± 2.41 vs. 4.68 ± 0.32; p = 0.048) and blood pressure (SBP and DBP; p ≤ 0.001) were observed (S1 Table).

Offspring ages varied between 2 years 8 months and 23 years 9 months, with similar sex distributions in the two groups (41% male in BMS vs. 43% in AMS; Table 1). BMS offspring were born 3 years 4 months (40.2 ± 28.0 months) before and AMS 3 years 9 months (44.9 ± 26.6 months) after maternal surgery. BMS offspring were older than AMS at follow-up (14.5 ± 5.7 vs. 9.0 ± 5.0 years; p = 0.001; BMS range: 5 years 9 months to 23 years 9 months; AMS range: 2 years 8 months to 19 years 6 months). Severe obesity was less prevalent in AMS using BMI percentile (p = 0.01) or BMI Z-score (p = 0.02). Adjusting for sex and puberty, AMS offspring exhibited trends toward lower fasting insulin levels and HOMA-IR index, and lower diastolic blood pressure (p < 0.10 for all).

**Table 1:** Offspring characteristics.

	**BMS**	**AMS**	**p-values** ^1^
N (males)	22 (9)	23 (10)	
Age (years)	14.5 ± 5.7	9.0 ± 5.0	0.001
Anthropometric data			
Fat percent^2^	29.6 ± 14.4	22.7 ± 10.3	0.28
BMI percentile	68.7 ± 41.5	69.7 ± 30.9	0.93
BMI Z-score^3^	1.93 ± 2.18	0.90 ± 1.48	0.08
Severe obesity			
BMI percentile > 98% (N)	11	3	0.01
BMI Z-score > 3 (N)	7	1	0.02
Blood pressure			
SBP (mm Hg)	110.5 ± 14.9	96.7 ± 14.8	0.13
DBP (mm Hg)	64.3 ± 10.5	52.8 ± 13.2	0.06
Lipid profile ^4^			
TG (mmol/l)	1.03 ± 0.44	0.81 ± 0.38	0.29
LDL-C (mmol/l)	2.66 ± 0.56	2.53 ± 0.59	0.67
HDL-C (mmol/l)	1.30 ± 0.31	1.30 ± 0.26	0.76
Total-C (mmol/l)	4.44 ± 0.67	4.20 ± 0.59	0.39
Total-C / HDL-C	3.58 ± 0.97	3.37 ± 0.87	0.76
Glucose metabolism ^4^			
Fasting glucose (mmol/l)	4.94 ± 0.44	4.77 ± 0.37	0.54
Insulin (μU/ml)	19.98 ± 12.54	11.45 ± 7.50	0.06
Homa-IR	4.55 ± 3.34	2.49 ± 1.74	0.08
CRP (mg/L)^5^	5.54 ± 8.34	1.54 ± 3.69	0.12

Values are presented as mean ± SD.

^1^ P-values adjusted for sex and puberty except for BMI percentile and BMI Z-score and obtained from comparison of all BMS (N = 22) and AMS (N = 23) offspring.

^2^ Fat percent at 6 years or more (BMS, N = 21; AMS, N = 15).

^3^ BMS, N = 19; AMS, N = 22.

^4^ BMS, N = 22; AMS, N = 20.

^5^ P-values for CRP were adjusted for the effects of sex, puberty and BMI percentile. Abbreviations: BMS, before maternal surgery; AMS, after maternal surgery; BMI, body mass index; SBP and DBP, systolic diastolic and systolic blood pressure; TG, triglycerides; LDL-C; low-density lipoprotein cholesterol; HDL-C, high-density lipoprotein cholesterol; Total-C, total cholesterol; CRP, C-reactive protein; SD, standard deviation.

### Interactions effects between offspring gene variations and maternal surgical status

Testing interactions between 2,550,297 offspring SNPs and maternal status on 16,060 expression phenotypes in offspring demonstrated 102,129 interactions reaching statistical significance (representing 0.00025% of all tests; p ≤ 1.22x10^–12^). The top significant interactions are shown in Table 2. These represent 48,156 unique SNPs (1.9% of the SNPs tested), 33.6% (16,184) of which demonstrated statistically significant interactions for multiple transcripts. Identified SNPs were mainly in intergenic regions (61%) while intronic, exonic and untranslated region SNPs represented a minority (35%, 2% and 2% respectively). SNP rs35447805 (kgp1360358) located at chr8:85131756 within the *RALYL* gene (NM_001100391) demonstrated the most statistically significant interaction with treatment for expression level of *SEPT4* (NM_080415) and *IFI35* (NM_005533), both located on chromosome 17 (Table 2). As an example, interaction effect for rs35447805 on *SEPT4* expression levels manifested in a 2.5 fold increase in expression for AMS heterozygous carriers and slight decrease (0.9 fold) for BMS heterozygous carriers (S2 Table). SNPs located in the interferon (alpha, beta and omega) receptor 1 (*IFNAR1*)—interleukin 10 receptor, beta (*IL10RB*) gene region and those near transmembrane protein, adipocyte associated 1 (GPR175, also known as TPRA1), BARX homeobox 2 (*BARX2*) transcription factor and heparan sulfate (glucosamine) 3-O-sulfotransferase 4 (*HS3ST4*) demonstrated several significant interactions (S3 Table).

**Table 2:** Most significant SNP-by-maternal treatment interactions.

**SNP**				**Transcript**			
**SNP ID** ^1^	**rs number**	**Chr**	**Position** ^2^	**Accession**	**Gene** ^3^	**Chr**	**P-value** ^4^
kgp11796917	rs146836802	14	30685942	NM_194323	OTOF	2	1.09x10^–57^
kgp30566776	rs73229805	X	67014865	NM_001010927	TIAM2	6	3.53x10^–45^
kgp8430951	rs78240927	6	163766098	NM_001100422	SPATS2L	2	2.37x10^–37^
kgp11155608	rs12059564	1	159000779	NM_015913	TXNDC12	1	7.81x10^–32^
kgp1360358	rs35447805	8	85131756	NM_080415	SEPT4	17	4.70x10^–30^
kgp6389054	rs78957751	16	34886264	NM_001006630	CHRM2	7	4.14x10^–27^
rs4764191	rs4764191	12	15560196	NM_001032295	SERPING1	11	4.43x10^–27^
kgp11709552	rs62227091	22	47811418	NM_001011724	HNRNPA1L2	13	3.15x10^–26^
kgp30620929	rs140547157	X	27936207	XM_945614	PMS2L1	7	3.32x10^–26^
kgp11042611	rs79592528	3	122772698	XM_938400	LOC142937	10	3.97x10^–26^
kgp8787288	rs940676	2	121235234	NM_003733	OASL	12	8.00x10^–25^
kgp4545030	rs78211484	12	59934484	CK299576	HS.528210	3	5.46x10^–24^
kgp18518	rs17757256	2	19650629	NM_001007524	F8A3	X	1.19x10^–23^
kgp2333358	rs1461092	12	41556961	NM_005419	STAT2	12	4.89x10^–23^
kgp8581526	rs36117773	5	11939359	NM_025126	RNF34	12	1.15x10^–22^
kgp494941	rs79270474	12	12338554	NM_002535	OAS2	12	1.59x10^–22^
kgp6448962	rs57573303	9	28322259	NM_001012978	BEX5	X	5.84x10^–22^
rs3803712	rs3803712	16	26074500	NM_001712	CEACAM1	19	7.21x10^–22^
kgp36317	rs2247335	6	106992592	NM_001007234	ERCC8	5	8.83x10^–22^
kgp4630305	rs59557850	8	78148122	NM_003728	UNC5C	4	1.10x10^–21^
rs9655226	rs9655226	7	22598742	NM_170695	TGIF1	18	3.07x10^–21^
kgp10592712	rs73063011	19	52338183	NM_024032	C17ORF53	17	8.71x10^–21^
kgp237322	rs539822	5	176310164	XM_001721497	LOC100132457	2	1.02x10^–20^
rs11053624	rs11053624	12	10283711	NM_015589	SAMD4A	14	1.51x10^–20^
kgp11540460	rs144298037	6	45434746	NM_001004349	FLJ45422	6	1.56x10^–20^
kgp11526978	rs4711691	6	12404895	NM_014453	CHMP2A	19	3.55x10^–20^
kgp11514400	rs35833993	6	165457847	NM_006918	SC5D	11	5.65x10^–20^
kgp3001132	rs9606166	22	19811720	NM_006704	SUGT1	13	6.90x10^–20^
kgp9305036	rs943009	6	11243891	NR_002940	LRRC37A4	17	1.59x10^–19^
kgp8789955	rs62576233	9	84337468	NM_005792	MPHOSPH6	16	1.63x10^–19^
kgp1173427	rs61733660	6	151148947	XM_925998	SRA1	5	1.82x10^–19^
rs2968402^5^	rs2968402^4^	16	21947480	NM_014598	SOCS7	17	3.16x10^–19^
kgp12304307	rs13298711	9	83820909	NM_002256	KISS1	1	3.45x10^–19^
rs11636802	rs11636802	15	56775597	DA276856	HS.576243	1	3.53x10^–19^
kgp12481432	rs4407201	2	130522894	NM_016134	CPQ	8	4.88x10^–19^
kgp31122632	rs138131809	X	116143012	NR_024524	LOC100129055	10	6.25x10^–19^
rs7149078	rs7149078	14	32844576	CD369504	HS.540642	16	6.57x10^–19^
kgp4554682	rs1108962	4	100663492	AI274046	HS.555512	14	8.86x10^–19^
kgp8947215	rs73030956	12	1675847	NM_018271	FLJ10916	2	9.40x10^–19^
kgp7059559	rs1831464	13	92902619	NM_019062	RNF186	1	9.67x10^–19^
kgp4609959	rs13284671	9	824742	NM_002720	PPP4C	16	1.02x10^–18^
kgp2913569	rs36067040	1	224238495	NM_001017977	DCAF6	1	1.32x10^–18^
kgp1360358	rs35447805	8	85131756	NM_005533	IFI35	17	1.67x10^–18^
kgp6643156	rs79321471	14	101498881	NM_017831	RNF125	18	1.81x10^–18^
kgp619464	rs4292995	1	30738298	NM_022148	CRLF2	Y	2.36x10^–18^
rs6074541	rs6074541	20	12978517	NM_006286	TFDP2	3	2.87x10^–18^
kgp12299095	rs35007051	2	188415764	NM_002164	IDO1	8	3.76x10^–18^
kgp12307971	rs76107005	10	85534815	NM_002201	ISG20	15	4.36x10^–18^
kgp10871570	rs115462216	21	22882926	U43604	HS.550193	5	4.78x10^–18^
rs3025651	rs3025651	6	29539914	NM_003646	DGKZ	11	6.03x10^–18^

List of the top 50 significant interactions. Regulated transcripts and respective p-values are shown.

SNP with the most significant association obtained from comparison of all BMS (N = 22) and AMS (N = 23) offspring was shown for each transcript.

^1^ SNP ID as defined by Illumina HumanOmni-5-Quad BeadChip annotation.

^2^ Genome build 37.

^3^ RefSeq or UniGene nomenclature.

^4^ P-values for differences between regression slopes (BMS vs. AMS) obtained from an additive model.

^5^ SNP mapped at two locations (chr16:21947480 and chr16:29119905). Abbreviations: SNP, single nucleotide polymorphism; Chr, chromosome.

Statistically significant offspring SNP-by-maternal surgical status interactions identified for the 48,156 unique SNPs involved 525 unique transcripts (3.3%) thus implying that a single transcript might be under multiple genetic constraints. Indeed, 375 of these 525 unique transcripts (71.4%) demonstrated multiple (≥2) significant interactions. The most highly represented transcripts from significant interactions are shown in Table 3, including transcripts encoding genes involved in regulation of gene expression *per se*, immune and inflammatory responses and lipid biosynthesis from which examples for *STAT2* (NM_005419), *IFI35* (NM_005533) and *DGKZ* (NM_003646) are shown in S1 Fig. LD was found between SNPs, resulting in multiple significant interactions with an identical transcript: 14,676 interaction tagging SNPs (tSNPs) were identified among the 48,156 unique SNPs showing significant interactions. Limiting analysis to these tSNPs led to the identification of 56.2% of the transcripts showing multiple statistically significant interactions.

**Table 3:** Most represented transcripts from the list of significant interactions.

**Transcript^1^**	**Accession**	**Significant interactions (N)**	**Most significant p-value^2^**
SEPT4	NM_080415	8492	4.70x10^–30^
OTOF	NM_194323	8473	1.09x10^–57^
SPATS2L	NM_001100422	8465	2.37x10^–37^
TXNDC12	NM_015913	8172	7.81x10^–32^
LOC142937	XM_938400	6690	3.97x10^–26^
HNRNPA1L2	NM_001011724	6151	3.15x10^–26^
HS.528210	CK299576	5804	5.46x10^–24^
TIAM2	NM_001010927	5204	3.53x10^–45^
OAS2	NM_002535	4396	1.59x10^–22^
SERPING1	NM_001032295	4168	4.43x10^–27^
OASL	NM_003733	2964	8.00x10^–25^
HS.550193	U43604	2886	4.78x10^–18^
FLJ45422	NM_001004349	2659	1.56x10^–20^
TCP1	NM_030752	1823	3.15x10^–17^
RNF125	NM_017831	1762	1.81x10^–18^
CEACAM1	NM_001712	1728	7.21x10^–22^
SRA1	XM_925998	1545	1.82x10^–19^
IFI35	NM_005533	1474	1.67x10^–18^
STAT2	NM_005419	1371	4.89x10^–23^
HS.391327	BX110374	1352	1.39x10^–16^
PMS2L1	XM_945614	1080	3.32x10^–26^
SAMD4A	NM_015589	949	1.51x10^–20^
DGKZ	NM_003646	840	6.03x10^–18^
ISG20	NM_002201	798	4.36x10^–18^
LOC649009	XM_941706	701	2.41x10^–17^
HS.553068	BX103476	533	1.32x10^–15^
F8A3	NM_001007524	502	1.19x10^–23^
BEX5	NM_001012978	475	5.84x10^–22^
LRRC37A4	NR_002940	462	1.59x10^–19^
LOC401525	XM_376869	447	8.58x10^–18^
LOC649143	XM_944822	403	6.06x10^–18^
SUGT1	NM_006704	400	6.90x10^–20^
FANCA	NM_000135	366	6.50x10^–18^
PPP4C	NM_002720	321	1.02x10^–18^
GOLM1	NM_177937	317	5.85x10^–17^
PIGC	NM_153747	295	4.51x10^–17^
TAPBP	NM_172209	274	1.56x10^–16^
ATF3	NM_001040619	216	3.66x10^–17^
RNF34	NM_025126	194	1.15x10^–22^
HS.539736	AI979341	192	9.90x10^–18^
LOC100132347	XM_001713703	186	2.06x10^–15^
LOC100132457	XM_001721497	168	1.02x10^–20^
CPQ	NM_016134	165	4.88x10^–19^
UBXD7	XM_936412	146	4.18x10^–15^
FAT3	XM_926199	145	1.15x10^–15^
RNF186	NM_019062	139	9.67x10^–19^
ZBP1	NM_030776	134	1.16x10^–14^
OPRL1	NM_000913	131	9.08x10^–15^
LOC645253	XM_944197	127	1.79x10^–15^
HIST1H4H	NM_003543	125	7.54x10^–16^

^1^ RefSeq or UniGene nomenclature.

^2^ P-values for differences between regression slopes (BMS vs. AMS) obtained from an additive model. Abbreviation: N, number.

### Gene functions and pathways

**Figure 1:**
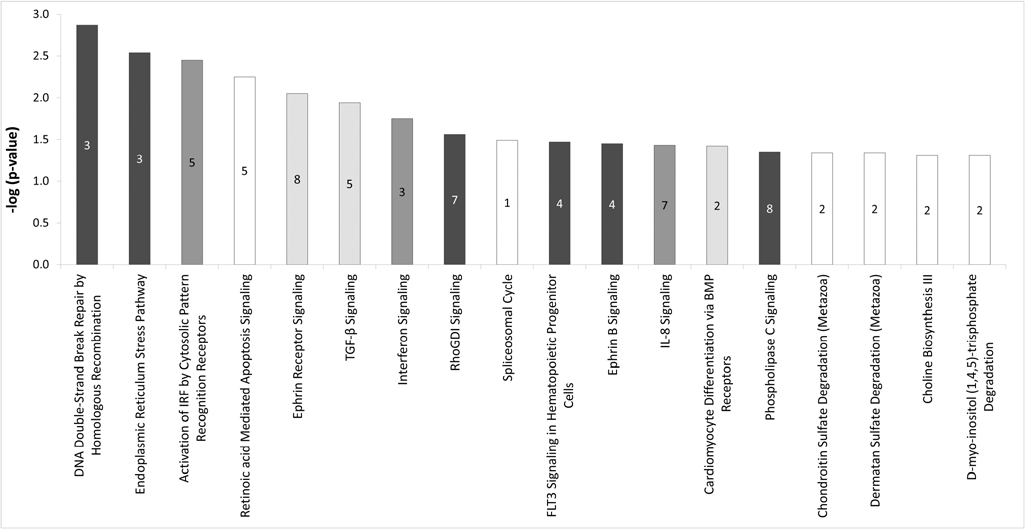
Pathways enriched from transcripts demonstrating significant interactions. The number of submitted transcripts in each pathway is reported in histogram bars. Histogram bars for pathways related to cellular stress and signaling (black), immune response and inflammation (dark grey), and growth, proliferation and development (light grey) are highlighted. Unrelated pathways are shown in white.

Clustering of the 525 transcripts showing significant interactions based on ontology using DAVID identified 5 over-represented functional categories (-log group enrichment score > 1.30; p-value for group enrichment score < 0.05): 1) transcription, 2) metabolic process, 3) guanine nucleotide exchange factor, 4) death/ZU5 domain and 5) zinc finger domain (S4 Table). IPA analysis revealed infectious disease, inflammatory response, gene expression, and cellular growth and proliferation among the over-represented functional categories from the list of transcripts demonstrating statistically significant interactions, thus highlighting transcription and cellular metabolism functions using both function analysis tools. Similarly, IPA revealed 18 pathways enriched for these transcripts, including 12 related to cellular stress and signaling, immune response and inflammation, and growth, proliferation and development. Importantly, DNA Double-Strand Break Repair by Homologous Recombination was the most overrepresented pathway (p = 0.001) and carbohydrate and lipid biosynthesis/degradation pathways were also identified (Fig. 1).

## Discussion

In a unique offspring cohort born discordant for maternal biliopancreatic bypass surgery affecting maternal metabolic fitness we extend observations that an adverse dysmetabolic intrauterine environment is associated with subsequent obesity and cardiometabolic risk [30, 31] related to gene expression levels [16, 18, 19]. Using a cohort in which gene expression and methylation levels were previously evaluated in regards to metabolic differences between BMS and AMS offspring [17, 32], the current study focusing on a different aspect (gene-environment interactions) demonstrated modulatory effects of maternal fitness on the association between genotype and gene expression in offspring. In this study we tested gene expression levels (expression traits) for interactions between offspring SNPs and maternal treatment. By analyzing the offspring genotypes and gene expression at the genome-wide level combined with the impact of maternal status, we provide an objective insight into the relation between maternal status and offspring cardiometabolic risk profile and have the potential to elucidate the functional basis of known associations identified previously.

Overlapping SNPs identified here with those previously associated with specific phenotypes and metabolic variables in GWAS has the potential to elucidate mechanisms for previously reported associations. Systematic comparison of SNPs and regulated transcripts with results from GWAS was then conducted. Our top interactions (Table 2) identified SNP rs57573303 in the gene *LINGO2* and SNP rs3803712 located in *HS3ST4*. The former was associated with *BEX5* expression levels, a brain expressed X-linked gene family member regulating differentiation of dopamine neurons involved in food reward signaling [44]. The latter we found was associated with *CEACAM1* gene expression levels encoding a cell-cell adhesion molecule involved in differentiation, apoptosis and modulation of innate and adaptive immune response consistent with decreased liver *CEACAM1* expression reported in severely obese patients [45]. In regards to the interaction identified here, increased *CEACAM1* expression was observed in heterozygous AMS offspring while BMS rare allele carriers demonstrated lower *CEACAM1* expression. SNPs in *LINGO2* and *HS3ST4* were previously found to be associated with children BMI z-score and % fat mass, respectively [11]. Associations between rs10968576, also in *LINGO2*, and fasting plasma cholesterol levels and BMI were found in different populations [46, 47]. We identify here rs115462216 SNP (kgp10871570) in *NCAM2* for which rs11088859 has been associated with waist circumference [48]. In addition, the presence of neurological (*ERCC8*, *KISS1*, *IDO1*, *OTOF*, *SEPT4*, *SERPING1*, *SOCS7*, *STAT2*, *TGIF1*, *TIAM2*) and endocrine system development (*CHRM2*, *SC5D*, *SOCS7*) genes among the regulated transcripts (Table 2) suggest a mechanistic role in offspring programming under varying maternal conditions [19, 49]. Taken together, the identification of SNPs located in genes previously reported by others to be associated with obesity traits support our results and is consistent with studies showing that SNPs associated with complex traits are more likely to be eQTLs [50, 51].

The limited number of transcripts for which we found offspring gene variation-by-maternal status interactions and the large number of SNPs suggest that single transcripts may be under multiple genetic constraints, coherent with previous studies conducted on larger cohorts [52–54]. Among the numerous SNPs we identified with significant interactions, many were specifically associated with the inflammatory, insulin-resistant, dysmetabolic diathesis of diabesity such as SNPs in the *IFNAR1—IL10RB* gene region forming the class II cytokine receptor gene cluster (S3 Table). Those genes involved in IL10-induced signal transductions were associated with inflammatory diseases and ischemic stroke with hypertension [55–57]. We also observed significant interactions for SNPs located near the *GPR175* (*TPRA1*) gene, expression level of which was previously demonstrated to be associated with plasma lipid levels [58]. Furthermore, we found interactions of SNPs near *BARX2*, a member of the homeobox transcription factor family known to influence cellular processes controlling cell adhesion and remodeling actin cytoskeleton. Others previously found such associations with T2DM and end-stage renal disease [59].

Gene function analysis conducted with two independent tools highlighted genes related to transcription and cellular metabolism. Combined with other overrepresented functional categories (guanine nucleotide exchange factor, death/ZU5 domain, zinc finger domain), these results suggested some potential effects of maternal metabolic fitness on offspring at both the cellular and transcriptional levels. Our pathway analysis also identified pathways related to cellular growth, proliferation and development, stress and signaling as well as carbohydrate and lipid metabolism similar to others’ findings. Global gene expression analysis of amniotic fluid cell-free fetal RNA identified lipid (*apolipoprotein D*) and transcriptional regulators (*FOS* and *STAT3*) as well as apoptotic cell death-related genes among differentially expressed genes between fetuses of obese vs. lean pregnant women [16]. In conjunction with influences of maternal obesity before conception reported on gene expression profiles of rat embryos, genes related to cell cycle, carbohydrate metabolism, DNA repair and transcriptional regulator were altered [60]. These results strengthen our observations pertaining to an overrepresentation of cellular processes (cellular growth, proliferation and development), carbohydrate and lipid metabolism pathways identified. The study on rat embryos [60] also supports overrepresentation of immune response and inflammatory genes as well as DNA double-strand break repair-related genes among transcripts with significant interactions. Similar to changes in expression previously observed in obese patients early after having undergone weight loss or bariatric surgery [26, 27, 29], we found that inflammation-related transcripts were overrepresented. Globally, overrepresentation of cellular signaling, carbohydrate metabolism and inflammatory pathways identified here from the list of transcripts showing significant offspring gene variations by maternal surgical status interactions are in line with previous results from our group comparing BMS and AMS offspring at gene methylation and expression levels [17, 32]. In addition, our results are consonant with murine studies showing effects of maternal gestational obesity and high-fat diet on offspring with differences in gene expression levels for genes related to inflammation and glucose homeostasis [61, 62] and for pathways related to cellular stress, signaling, growth, proliferation, development and regulation of lipogenic pathways [63] of significant pathogenic importance for the dysmetabolic diathesis of diabesity. Our pathway analysis demonstrated involvement of carbohydrate and lipid metabolism pathways, in agreement with results from maternal weight loss studies in sheep demonstrating an impact on insulin signaling, glucose transport and glycogen synthesis pathways in offspring’ skeletal muscle [64] as well as with previous studies demonstrating gene-by-maternal diet interactions in offspring [21, 65].

Some of the modulating effects of gestational metabolic fitness may be confounded. Young age of the offspring limits the potential contribution of different postnatal environments. However, it does not allow extrapolation over the life span stretching into mature adulthood when most pathology emerges through the cumulative effects of environmental exposures. Nevertheless, the preponderance of literature on developmental origins of adult disease, specifically for cardio-metabolic outcomes related to our findings demonstrates durability of effects over the life-span [15]. The rarity of gastrointestinal biliopancreatic bypass surgery, low pregnancy rates before and after maternal surgery, constraints of study design and the exclusive nature of the molecular analyses all limited the size of our offspring population. The size of the study sample limited the number of adjustments made to correct for confounding factors relating to offspring and maternal condition during pregnancy (breastfeeding, smoking, etc.). Adjustments for confounding factors were thus limited to sex and puberty. Although metabolic parameters in the offspring cohorts were not statistically significantly different owing to sample size, the differences were clinically significant particularly for insulin resistance, dyslipidemia and CRP. Studies from our group conducted on larger cohorts have previously demonstrated robust group differences between BMS and AMS offspring [30, 31]. Our gene analyses were performed on blood, more convenient to obtain and to justify sampling than other tissues in healthy juvenile offspring. We and others have reported partial inter-tissue correlations [54, 66–68]. The multicellular nature of blood constitutes an inherent limitation in our study; tissue heterogeneity potentially influenced measurement of gene expression levels [69, 70]. Nonetheless, we assessed gene expression as representative of systemic biological differences between BMS and AMS offspring to which multiple organs and tissues have contributed. Causality of identified variants cannot be determined: identified variants might be markers of genomic regions or loci in which causal variants lie and allelic heterogeneity cannot be ruled out, together necessitating much larger population studies than our unique but relatively small cohort study.

Strengths of our study are the unique genetically and phenotypically characterized offspring cohort discordant for maternal gestational metabolic fitness, the efficacious standardized currently performed metabolic operation as a tool to alter the intrauterine milieu and an exceptionally high follow-up rate enabled by the national health insurance system. The biliopancreatic bypass operation selectively increases steatorrhea, lowering maternal plasma free fatty acids, reducing fatty infiltration of metabolically active tissues, reducing lipid peroxidation and systemic lipotoxic inflammation altogether improving insulin action and glucose disposal approximating pre-obese levels. Although these durable effects were not replicated quantitatively or qualitatively by other current bariatric operations, they add critical insight into molecular mechanisms associated with the gene expression levels presented here.

Our results demonstrated influences of the intrauterine metabolic environment on associations between offspring genotype and gene expression levels. The lower prevalence of obesity and cardiometabolic risk observed in AMS offspring argues for implementation of maternal weight loss and improved metabolic fitness before pregnancy and provides potential mechanisms for physiological improvements through regulation of gene expression in offspring.

## Supporting Information

S1 Fig Example of transcripts under multiple genetic constraints from the most represented transcripts. Panel A, *STAT2* (NM_005419). Panel B, *IFI35* (NM_005533). Panel C, *DGKZ* (NM_003646).
(TIF)

S1 Table Mothers’ characteristics. (DOCX)

S2 Table Gene expression levels for most significant SNP-by-maternal status interactions. SNPs, regulated transcripts and genotype-specific gene expression levels are shown. Expression values (means) relative to common homozygotes from the BMS group.
(DOCX)

S3 Table Most represented SNPs from the list of significant interactions. (DOCX)

S4 Table Functional clusters for transcripts with significant associations. (DOCX)
