# Supplementary figures and images for: Influences of Gestational Obesity on Associations between Genotypes and Gene Expression Levels in Offspring following Maternal Gastrointestinal Bypass Surgery for Obesity
Source: PLoS One. 2015 Jan 20;10(1):e0117011. doi: 10.1371/journal.pone.0117011 (PMC4300091; doi:10.1371/journal.pone.0117011)

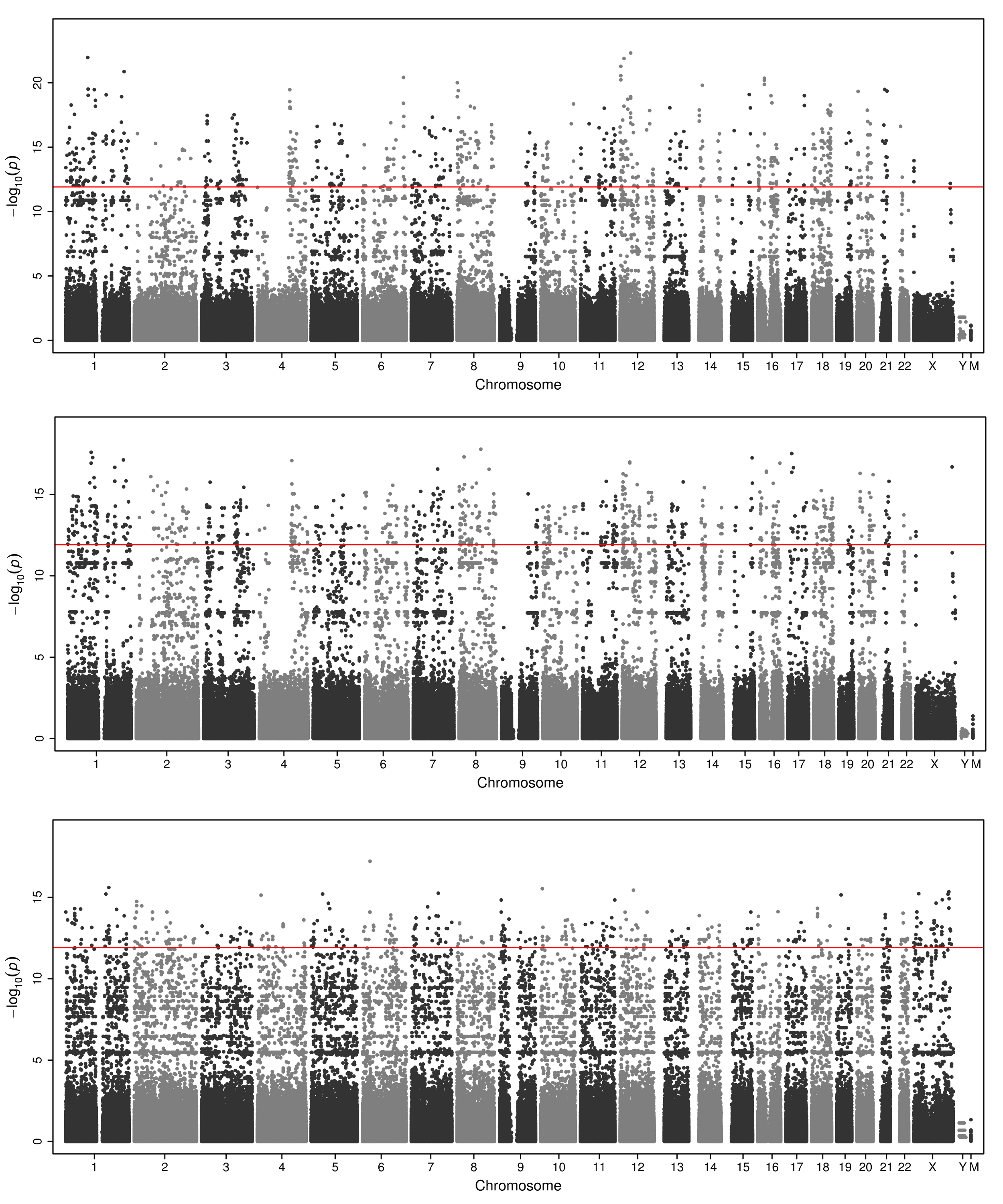

Supplement: S1 Fig — Panel A, STAT2 (NM_005419). Panel B, IFI35 (NM_005533). Panel C, DGKZ (NM_003646). (TIF) [file pone.0117011.s001.tif]
