# Supplementary material for: Influences of Gestational Obesity on Associations between Genotypes and Gene Expression Levels in Offspring following Maternal Gastrointestinal Bypass Surgery for Obesity
Source: PLoS One. 2015 Jan 20;10(1):e0117011. doi: 10.1371/journal.pone.0117011 (PMC4300091; doi:10.1371/journal.pone.0117011)
Supplement: S1 Table — (DOCX) [file pone.0117011.s002.docx]

**Supplementary Table S1. Mothers’ characteristics.**

|  | **Pre-surgical** | **Postoperative** | **p-value** |
| --- | --- | --- | --- |
| Age (years) | 29.5 ± 4.2 | 41.4 ± 5.2 | < 0.0001 |
| Anthropometric data |  |  |  |
| Weight (kg) | 121.6 ± 18.7 | 74.9 ± 12.2 | < 0.0001 |
| Height (m) | 164.4 ± 5.3 | 164.9 ± 6.7 | 0.3 |
| BMI | 45.1 ± 7.4 | 27.6 ± 4.9 | < 0.0001 |
| Blood pressure |  |  |  |
| SBP (mm Hg) | 137.4 ± 11.7 | 112.3 ± 9.8 | < 0.0001 |
| DBP (mm Hg) | 88.4 ± 10.9 | 69.7 ± 14.6 | 0.001 |
| Lipid profile |  |  |  |
| TG (mmol/l) | 1.64 ± 0.79 | 0.98 ± 0.42 | 0.005 |
| LDL-C (mmol/l) | 2.97 ± 0.85 | 1.65 ± 0.50 | < 0.0001 |
| HDL-C (mmol/l) | 1.13 ± 0.26 | 1.39 ± 0.25 | < 0.0001 |
| Total-C (mmol/l) | 4.89 ± 0.80 | 3.49 ± 0.49 | < 0.0001 |
| Total-C / HDL-C | 3.33 ± 1.09 | 2.59 ± 0.60 | 0.002 |
| Glucose metabolism |  |  |  |
| Fasting glucose (mmol/l) | 5.81 ± 2.41 | 4.68 ± 0.32 | 0.048 |
| Insulin (μU/ml)^1^ | 42.2 ± 30.9 | 8.7 ± 2.2 | 0.20 |
| Homa-IR^1^ | 9.1 ± 7.7 | 1.8 ± 0.5 | 0.24 |

Values are presented as mean ± SD. ^1^ Pre-surgical insulin levels and Homa-IR index were only available for 3 women. Abbreviations: BMI, body mass index; SBP and DBP, systolic diastolic and systolic blood pressure; TG, triglycerides; LDL-C; low-density lipoprotein cholesterol; HDL-C, high-density lipoprotein cholesterol; Total-C, total cholesterol; SD, standard deviation.
