# Supplementary material for: Influences of Gestational Obesity on Associations between Genotypes and Gene Expression Levels in Offspring following Maternal Gastrointestinal Bypass Surgery for Obesity
Source: PLoS One. 2015 Jan 20;10(1):e0117011. doi: 10.1371/journal.pone.0117011 (PMC4300091; doi:10.1371/journal.pone.0117011)
Supplement: S2 Table — SNPs, regulated transcripts and genotype-specific gene expression levels are shown. Expression values (means) relative to common homozygotes from the BMS group. (DOCX) [file pone.0117011.s003.docx]

**Supplementary Table S2. Gene expression levels for most significant SNP-by-maternal status interactions.** SNPs, regulated transcripts and genotype-specific gene expression levels are shown. Expression values (means) relative to common homozygotes from the BMS group.

| **SNP** |  | **Transcript** |  |  | **BMS** |  |  |  | **AMS** |  |  |
| --- | --- | --- | --- | --- | --- | --- | --- | --- | --- | --- | --- |
| **SNP ID^1^** |  | **Accession** | **Gene^2^** |  | Common HMZ | HTZ | Rare HMZ |  | Common HMZ | HTZ | Rare HMZ |
| kgp11796917 |  | NM_194323 | OTOF |  | 1.00 | 0.87 | --- |  | 0.95 | 2.75 | --- |
| kgp30566776 |  | NM_001010927 | TIAM2 |  | 1.00 | 0.83 | 0.67 |  | 0.77 | 2.13 | --- |
| kgp8430951 |  | NM_001100422 | SPATS2L |  | 1.00 | 0.98 | --- |  | 1.02 | 2.76 | --- |
| kgp11155608 |  | NM_015913 | TXNDC12 |  | 1.00 | 1.04 | 1.04 |  | 1.03 | 0.59 | --- |
| kgp1360358 |  | NM_080415 | SEPT4 |  | 1.00 | 0.90 | --- |  | 0.97 | 2.48 | --- |
| kgp6389054 |  | NM_001006630 | CHRM2 |  | 1.00 | 1.63 | --- |  | 1.11 | 0.98 | 0.95 |
| rs4764191 |  | NM_001032295 | SERPING1 |  | 1.00 | 0.69 | 0.73 |  | 0.84 | 2.77 | --- |
| kgp11709552 |  | NM_001011724 | HNRNPA1L2 |  | 1.00 | 1.90 | --- |  | 1.05 | 0.96 | 1.04 |
| kgp30620929 |  | XM_945614 | PMS2L1 |  | 1.00 | 0.96 | --- |  | 0.95 | 1.65 | --- |
| kgp11042611 |  | XM_938400 | LOC142937 |  | 1.00 | 1.84 | --- |  | 1.08 | 0.98 | --- |
| kgp8787288 |  | NM_003733 | OASL |  | 1.00 | 0.81 | --- |  | 0.88 | 2.32 | --- |
| kgp4545030 |  | CK299576 | HS.528210 |  | 1.00 | 1.85 | --- |  | 1.01 | 1.04 | 0.98 |
| kgp18518 |  | NM_001007524 | F8A3 |  | 1.00 | 1.82 | --- |  | 0.99 | 0.88 | 1.02 |
| kgp2333358 |  | NM_005419 | STAT2 |  | 1.00 | 0.95 | --- |  | 1.01 | 1.25 | --- |
| kgp8581526 |  | NM_025126 | RNF34 |  | 1.00 | 1.75 | --- |  | 1.01 | 0.94 | 1.00 |
| kgp494941 |  | NM_002535 | OAS2 |  | 1.00 | 0.90 | --- |  | 1.03 | 2.79 | --- |
| kgp6448962 |  | NM_001012978 | BEX5 |  | 1.00 | 1.65 | --- |  | 1.01 | 0.95 | 0.84 |
| rs3803712 |  | NM_001712 | CEACAM1 |  | 1.00 | 0.82 | 0.91 |  | 0.85 | 1.93 | --- |
| kgp36317 |  | NM_001007234 | ERCC8 |  | 1.00 | 1.53 | --- |  | 1.05 | 1.00 | 0.96 |
| kgp4630305 |  | NM_003728 | UNC5C |  | 1.00 | 0.88 | --- |  | 0.87 | 1.72 | --- |
| rs9655226 |  | NM_170695 | TGIF1 |  | 1.00 | 0.85 | --- |  | 0.80 | 0.90 | 0.94 |
| kgp10592712 |  | NM_024032 | C17ORF53 |  | 1.00 | 0.91 | 0.79 |  | 0.97 | 1.49 | --- |
| kgp237322 |  | XM_001721497 | LOC100132457 |  | 1.00 | 1.70 | --- |  | 1.14 | 1.02 | 0.92 |
| rs11053624 |  | NM_015589 | SAMD4A |  | 1.00 | 0.90 | --- |  | 0.88 | 1.84 | --- |
| kgp11540460 |  | NM_001004349 | FLJ45422 |  | 1.00 | 1.68 | --- |  | 0.98 | 0.91 | --- |
| kgp11526978 |  | NM_014453 | CHMP2A |  | 1.00 | 0.76 | --- |  | 0.95 | 1.00 | --- |
| kgp11514400 |  | NM_006918 | SC5D |  | 1.00 | 1.32 | 1.71 |  | 1.06 | 1.02 | 1.04 |
| kgp3001132 |  | NM_006704 | SUGT1 |  | 1.00 | 0.62 | 0.81 |  | 0.91 | 1.63 | --- |
| kgp9305036 |  | NR_002940 | LRRC37A4 |  | 1.00 | 0.83 | --- |  | 0.90 | 1.68 | --- |
| kgp8789955 |  | NM_005792 | MPHOSPH6 |  | 1.00 | 1.32 | --- |  | 1.12 | 0.98 | --- |
| kgp1173427 |  | XM_925998 | SRA1 |  | 1.00 | 0.99 | --- |  | 0.98 | 1.00 | --- |
| rs2968402^3^ |  | NM_014598 | SOCS7 |  | 1.00 | 1.41 | --- |  | 1.06 | 1.00 | --- |
| kgp12304307 |  | NM_002256 | KISS1 |  | 1.00 | 0.96 | 0.90 |  | 0.94 | 1.47 | --- |
| rs11636802 |  | DA276856 | HS.576243 |  | 1.00 | 1.80 | --- |  | 1.08 | 0.93 | --- |
| kgp12481432 |  | NM_016134 | CPQ |  | 1.00 | 1.02 | --- |  | 0.99 | 0.87 | --- |
| kgp31122632 |  | NR_024524 | LOC100129055 |  | 1.00 | 1.94 | --- |  | 1.12 | 0.89 | 1.05 |
| rs7149078 |  | CD369504 | HS.540642 |  | 1.00 | 0.65 | --- |  | 0.73 | 0.71 | 0.74 |
| kgp4554682 |  | AI274046 | HS.555512 |  | 1.00 | 0.70 | 0.58 |  | 0.73 | 0.75 | 0.71 |
| kgp8947215 |  | NM_018271 | FLJ10916 |  | 1.00 | 0.66 | 1.03 |  | 0.91 | 1.53 | --- |
| kgp7059559 |  | NM_019062 | RNF186 |  | 1.00 | 0.90 | --- |  | 1.00 | 1.47 | --- |
| kgp4609959 |  | NM_002720 | PPP4C |  | 1.00 | 0.84 | --- |  | 0.96 | 0.98 | 1.01 |
| kgp2913569 |  | NM_001017977 | DCAF6 |  | 1.00 | 1.78 | --- |  | 1.10 | 0.98 | 0.94 |
| kgp1360358 |  | NM_005533 | IFI35 |  | 1.00 | 0.94 | --- |  | 0.98 | 1.42 | --- |
| kgp6643156 |  | NM_017831 | RNF125 |  | 1.00 | 0.84 | 0.99 |  | 0.94 | 1.86 | --- |
| kgp619464 |  | NM_022148 | CRLF2 |  | 1.00 | 0.91 | 0.93 |  | 0.89 | 1.37 | --- |
| rs6074541 |  | NM_006286 | TFDP2 |  | 1.00 | 0.88 | 0.92 |  | 0.94 | 1.31 | --- |
| kgp12299095 |  | NM_002164 | IDO1 |  | 1.00 | 0.81 | --- |  | 0.92 | 2.40 | --- |
| kgp12307971 |  | NM_002201 | ISG20 |  | 1.00 | 0.97 | --- |  | 0.99 | 1.19 | --- |
| kgp10871570 |  | U43604 | HS.550193 |  | 1.00 | 1.97 | --- |  | 1.05 | 0.89 | 0.79 |
| rs3025651 |  | NM_003646 | DGKZ |  | 1.00 | 1.89 | --- |  | 1.03 | 0.83 | --- |

^1^ SNP ID as defined by Illumina HumanOmni-5-Quad BeadChip annotation. SNP with the most significant association was shown for each transcript. ^2^ RefSeq or UniGene nomenclature. ^3^ SNP mapped at two locations (chr16:21947480 and chr16:29119905). Abbreviations: SNP, single nucleotide polymorphism; BMS, before maternal surgery; AMS, after maternal surgery; HMZ, homozygotes; HTZ, heterozygotes.
