# Supplementary material for: Influences of Gestational Obesity on Associations between Genotypes and Gene Expression Levels in Offspring following Maternal Gastrointestinal Bypass Surgery for Obesity
Source: PLoS One. 2015 Jan 20;10(1):e0117011. doi: 10.1371/journal.pone.0117011 (PMC4300091; doi:10.1371/journal.pone.0117011)
Supplement: S3 Table — (DOCX) [file pone.0117011.s004.docx]

**Supplementary Table S3. Most represented SNPs from the list of significant interactions.**

| **SNP ID^1^** | **rs number** | **Chr** | **Position^2^** | **Nearest gene^3^** | **Localization** | **Interactions (N)** |
| --- | --- | --- | --- | --- | --- | --- |
| kgp11076368 | rs2834192 | 21 | 34704102 | IFNAR1 | Intron | 18 |
| kgp5498146 | rs17875779 | 21 | 34703703 | IFNAR1 | Intron | 18 |
| kgp8643118 | rs41349446 | 21 | 34689690 | IL10RB \| IFNAR1 | Intergenic | 18 |
| rs1041429 | rs1041429 | 21 | 34699909 | IFNAR1 | Intron | 18 |
| rs17643419 | rs17643419 | 21 | 34711798 | IFNAR1 | Intron | 18 |
| rs17875789 | rs17875789 | 21 | 34705711 | IFNAR1 | Intron | 18 |
| rs17875795 | rs17875795 | 21 | 34706773 | IFNAR1 | Intron | 18 |
| rs2834191 | rs2834191 | 21 | 34696502 | IL10RB \| IFNAR1 | Intergenic | 18 |
| rs7277852 | rs7277852 | 21 | 34679203 | IL10RB \| IFNAR1 | Intergenic | 18 |
| rs8118745 | rs8118745 | 20 | 40679500 | CHD6 \| PTPRT | Intergenic | 18 |
| rs8178556 | rs8178556 | 21 | 34666555 | IL10RB | Intron | 18 |
| kgp10535972 | rs2720233 | 3 | 127244252 | C3orf56 \| GPR175 | Intergenic | 17 |
| kgp10880723 | rs61911226 | 11 | 129296124 | BARX2 | Intron | 17 |
| kgp10946249 | rs73285847 | 12 | 30149831 | TMTC1 \| IPO8 | Intergenic | 17 |
| kgp11391174 | rs75662697 | 7 | 95253005 | PDK4 \| LOC100287137 | Intergenic | 17 |
| kgp12112667 | rs57076671 | 11 | 129298917 | BARX2 | Intron | 17 |
| kgp12126163 | rs7966489 | 12 | 3114018 | TEAD4 | Intron | 17 |
| kgp1360358 | rs35447805 | 8 | 85131756 | RALYL | Intron | 17 |
| kgp1773977 | rs77197461 | 11 | 129302906 | BARX2 | Intron | 17 |
| kgp1782156 | rs77197461 | 16 | 26081757 | HS3ST4 \| C16orf82 | Intergenic | 17 |
| kgp3920605 | rs72965105 | 1 | 88417463 | LMO4 \| PKN2 | Intergenic | 17 |
| kgp4992477 | rs79677673 | 16 | 26069225 | HS3ST4 \| C16orf82 | Intergenic | 17 |
| kgp5175422 | rs75400601 | 12 | 3119224 | TEAD4 | Intron | 17 |
| kgp6183971 | rs61617510 | 11 | 129299733 | BARX2 | Intron | 17 |
| kgp6424178 | rs2335331 | 12 | 3117015 | TEAD4 | Intron | 17 |
| kgp8814136 | rs72669821 | 14 | 33187426 | AKAP6 | Intron | 17 |
| rs10848760 | rs10848760 | 12 | 3096134 | TEAD4 | Intron | 17 |
| rs11053624 | rs11053624 | 12 | 10283711 | CLEC7A \| OLR1 | Intergenic | 17 |
| rs11062447 | rs11062447 | 12 | 3105007 | TEAD4 | Intron | 17 |
| rs11609673 | rs11609673 | 12 | 3113401 | TEAD4 | Intron | 17 |
| rs12312282 | rs12312282 | 12 | 3102446 | TEAD4 | Intron | 17 |
| rs2068970 | rs2068970 | 12 | 3107745 | TEAD4 | Intron | 17 |
| rs241362 | rs241362 | 1 | 229004264 | RHOU \| RAB4A | Intergenic | 17 |
| rs3803712 | rs3803712 | 16 | 26074500 | HS3ST4 \| C16orf82 | Intergenic | 17 |
| rs4764191 | rs4764191 | 12 | 15560196 | PTPRO | Intron | 17 |
| kgp1067116 | rs319346 | 7 | 95576363 | DYNC1I1 | Intron | 16 |
| kgp11155608 | rs12059564 | 1 | 159000779 | IFI16 | Intron | 16 |
| kgp1890902 | rs61781084 | 1 | 92244210 | TGFBR3 | Intron | 16 |
| kgp2178574 | rs11893686 | 2 | 82937695 | LOC100289658 \| LOC388965 | Intergenic | 16 |
| kgp22789029 | rs35589108 | 6 | 160207845 | TCP1 | Intron | 16 |
| kgp2880301 | rs1872214 | 12 | 30148566 | TMTC1 \| IPO8 | Intergenic | 16 |
| kgp3468696 | rs11656969 | 17 | 72311177 | DNAI2 \| KIF19 | Intergenic | 16 |
| kgp3810432 | rs61781086 | 1 | 92247533 | TGFBR3 | Intron | 16 |
| kgp4804673 | rs112723259 | 2 | 66795906 | MEIS1 | Intron | 16 |
| kgp5115620 | rs1508987 | 4 | 127374198 | FAT4 \| LOC729424 | Intergenic | 16 |
| kgp5517929 | rs66792294 | 3 | 120083994 | LOC100130701 | Intron | 16 |
| kgp6412082 | rs13217003 | 6 | 160315832 | PNLDC1 \| MAS1 | Intergenic | 16 |
| kgp7935811 | rs73285852 | 12 | 30150864 | TMTC1 \| IPO8 | Intergenic | 16 |
| kgp9192933 | rs35057779 | 1 | 87085961 | CLCA4 \| CLCA3P | Intergenic | 16 |
| rs11466561 | rs11466561 | 1 | 92314842 | TGFBR3 | Intron | 16 |
| rs1482923 | rs1482923 | 3 | 126920448 | C3orf56 \| GPR175 | Intergenic | 16 |
| rs4679349 | rs4679349 | 3 | 126917584 | C3orf56 \| GPR175 | Intergenic | 16 |
| rs4787345 | rs4787345 | 16 | 26082145 | HS3ST4 \| C16orf82 | Intergenic | 16 |
| rs512589 | rs512589 | 1 | 218708110 | TGFB2 \| LYPLAL1 | Intergenic | 16 |
| rs900419 | rs900419 | 3 | 126926547 | C3orf56 \| GPR175 | Intergenic | 16 |

^1^ SNP ID as defined by Illumina HumanOmni-5-Quad BeadChip annotation. ^2^ Genome build 37. ^3^ RefSeq or UniGene nomenclature. Abbreviations: Chr, chromosome; N, number; SNP, single nucleotide polymorphism.
