# Supplementary material for: Influences of Gestational Obesity on Associations between Genotypes and Gene Expression Levels in Offspring following Maternal Gastrointestinal Bypass Surgery for Obesity
Source: PLoS One. 2015 Jan 20;10(1):e0117011. doi: 10.1371/journal.pone.0117011 (PMC4300091; doi:10.1371/journal.pone.0117011)
Supplement: S4 Table — (DOCX) [file pone.0117011.s005.docx]

**Supplementary Table S4. Functional clusters for transcripts with significant associations.**

| **Terms** | **Enrichment Score** | **Minimum DAVID p-value** | **Number of genes** |
| --- | --- | --- | --- |
| Transcription | 1.82 | 0.001 | 22 |
| Metabolic process | 1.81 | 0.001 | 6 |
| Guanine nucleotide exchange factor | 1.78 | 0.0002 | 8 |
| Death/ZU5 domain | 1.60 | 0.01 | 3 |
| Zinc finger domain | 1.38 | 0.02 | 4 |

Enrichment score ranks the biological significance of terms on mean overall p-values of enriched annotation terms.
